# Challenges in tolerogenic dendritic cell therapy for autoimmune diseases: the route of administration

**DOI:** 10.1093/immadv/ltad012

**Published:** 2023-07-18

**Authors:** María José Mansilla, Catharien M U Hilkens, Eva M Martínez-Cáceres

**Affiliations:** Division of Immunology, LCMN, Germans Trias i Pujol University Hospital and Research Institute, Campus Can Ruti, Badalona, Spain; Department of Cellular Biology, Physiology and Immunology, Universitat Autònoma de Barcelona, 08193 Bellaterra (Cerdanyola del Vallès), Spain; Translational and Clinical Research Institute, Newcastle University, Newcastle upon Tyne, UK; Division of Immunology, LCMN, Germans Trias i Pujol University Hospital and Research Institute, Campus Can Ruti, Badalona, Spain; Department of Cellular Biology, Physiology and Immunology, Universitat Autònoma de Barcelona, 08193 Bellaterra (Cerdanyola del Vallès), Spain

**Keywords:** tolerogenic dendritic cells, route of administration, autoimmunity, cell therapy, immune tolerance

## Abstract

Tolerogenic dendritic cells (tolDCs) are a promising strategy to treat autoimmune diseases since they have the potential to re-educate and modulate pathological immune responses in an antigen-specific manner and, therefore, have minimal adverse effects on the immune system compared to conventional immunosuppressive treatments. TolDC therapy has demonstrated safety and efficacy in different experimental models of autoimmune disease, such as multiple sclerosis (MS), type 1 diabetes (T1D), and rheumatoid arthritis (RA). Moreover, data from phase I clinical trials have shown that therapy with tolDCs is safe and well tolerated by MS, T1D, and RA patients. Nevertheless, various parameters need to be optimized to increase tolDC efficacy. In this regard, one important parameter to be determined is the most appropriate route of administration. Several delivery routes, such as intravenous, subcutaneous, intraperitoneal, intradermal, intranodal, and intraarticular routes, have been used in experimental models as well as in phase I clinical trials. This review summarizes data obtained from preclinical and clinical studies of tolDC therapy in the treatment of MS, T1D, and RA and their animal models, as well as data from the context of cancer immunotherapy using mature peptide-loaded DC, and data from *in vivo* cell tracking experiments, to define the most appropriate route of tolDC administration in relation to the most feasible, safest, and effective therapeutic use.

## Introduction

Tolerogenic dendritic cells (tolDCs) are described as semimature or maturation-resistant DCs exhibiting the tolerogenic functionality of immature DCs (iDCs) as well as some properties of mature DCs, such as antigen presentation to T cells, migration to lymphoid organs and functional stability. TolDCs are characterized by the expression of low levels of costimulatory molecules (CD: cluster of differentiation [CD40, CD80, and CD86]) and major histocompatibility complex (MHC) class II, the expression of inhibitory molecules (programmed death ligand 1 [PDL1], immunoglobulin-like transcript 3 [ILT3], and immunoglobulin-like transcript 4 [ILT4]), the production of immunoregulatory mediators and cytokines (tumor growth factor [TGF]-β, interleukin [IL]-10, indoleamine 2 3-dioxygenase [IDO], and Fas ligand [FasL]) and decreased production of proinflammatory cytokines. Importantly, tolDCs induce T-cell hyporesponsiveness by promoting T-cell anergy, T-cell depletion, or regulatory T-cell (Treg) differentiation. Therefore, the use of tolDCs to restore long-lasting immune tolerance is a promising strategy targeting the origin of autoimmune disorders such as multiple sclerosis (MS), type 1 diabetes (T1D), and rheumatoid arthritis (RA).

In this context, multiple protocols for *ex vivo* tolDC generation from autologous human peripheral blood monocytes have been described: treatment with pharmacological and immunosuppressive agents such as vitamin D3 (VitD3), dexamethasone (Dexa), rapamycin or nuclear factor-kappa B (NF-κB) inhibitor (Bay 11-7082); culture in the presence of anti-inflammatory cytokines (IL-10 and TGF-β); or genetic engineering for the specific inhibition or induction of key molecules and pathways (such as the downregulation of CD40, CD80, and CD86 costimulatory molecules). A crucial step in tolDC manufacturing is their stability after exposure to proinflammatory conditions. For this reason, most of the tolDC manufacturing protocols include a maturation step using compounds such as lipopolysaccharide (LPS), monophosphoryl lipid A (MPLA), IL-1β, TNF-α, prostaglandin E2 (PGE2), or IL-6 to induce an activation-resistant state in tolDCs. Furthermore, this maturation stimulus triggers C-C chemokine receptor 7 (CCR7) expression, which is necessary for tolDC migration to the lymph nodes.

The success of tolDC therapy in autoimmune diseases is dependent on, among other factors, their effective migration to the lymph nodes or inflamed tissues to induce long-lasting immunoregulatory responses. In this context, the optimization of the tolDCs delivery route is critical for evaluating their efficacy in phase II clinical trials. This review analyses the clinical effects and routes of administration from animal models and clinical trials using therapeutic tolDCs in the treatment of autoimmune disorders.

## Current knowledge from preclinical and clinical studies

Despite the promising potential of tolDCs to restore therapeutic antigen-specific tolerance in patients suffering from autoimmune disorders, important questions regarding the best dose and route of delivery remain unanswered. These questions are particularly challenging because, in contrast to pharmacological drugs, tolDC treatment is a cell therapy that exerts its therapeutic effect via complex and not completely understood mechanisms.

This review analyses data from preclinical and clinical studies using tolDCs in the treatment of MS, T1D, and RA and their respective animal models, experimental autoimmune encephalomyelitis (EAE), nonobese diabetes (NOD), and collagen-induced arthritis (CIA) (Tables 1 and 2).

**Table 1.** Experimental models using tolDC treatment in a therapeutic approach

EAE model										
Route of administration	Dose	Number of administrations and interval of dose	EAE model	tolDC therapy			Type of treatment	Outcome	Observations	Ref
				Tolerogenic agent	Antigen	Maturation				
Intravenous	0.3 × 10^6^	3 administrations every 4 days (11, 14, and 17 pi)	C57Bl/6-MOG_35-55_	LPS	MOG_35-55_	LPS at 1 µg/ml for 24 h	Early therapeutic treatment	MOG-LPS-DC treatment inhibited EAE development by inducing percentage of Treg (CD4^+^CD25^+^FoxP3^+^GITR^+^) that are CD127^+^ 3G11^-^ and reducing CD127^+^ 3G11^+^ Treg		[1]
				Apoptotic thymocytes	MOG_35-55_	No	Early therapeutic treatment	MOG-specific DC primed with irradiated (apoptotic) T cells abrogate EAE development by inhibiting CD4^+^ effector memory T cells and IFNγ production	Splenic CD11c+ DCs instead of BMDCs were used	[2]
	0.8 × 10^6^	3 administrations every 3 days (10, 13, and 16 pi)	C57Bl/6-MOG_35-55_	VitD3	MOG_35-55_	No	Early therapeutic treatment	Delay of disease onset and clinical amelioration. Increase proportions of Treg, CD4+ IL‐10+ T cells and Breg in spleen and/or lymph nodes and reduced infiltration of Th1 and Th17 cells into spinal cord	Enhanced proportions of Th1 and Th17 cells in spleen and lymph nodes and increased level of IL-17 in serum	[4]
	1 × 10^6^	1 single administration at day 10 pi	C57Bl/6-MOG_35-55_	Engineered DCs to overexpress 25-hydroxyvitamin D 1α-hydroxylase	MOG_35-55_	LPS at 100 ng/ml	Early therapeutic treatment	Myelin-specific suppression of ongoing EAE symptoms related with induction of FoxP3 Treg and reduction of inflammation and demyelination in the spinal cord		[5]
		3 administrations every 3 days (days 10, 13, and 16 pi)	C57Bl/6-MOG_35-55_	K313	MOG_35-55_	LPS at 100 ng/ml for 24 h	Early therapeutic treatment	Reduction of clinical severity, CNS infiltration and demyelination. Increased frequency of Treg and reduction of Th1 and Th17 cells in the spleen	Similar clinical benefit of antigen-specific VitD3-tolDCs	[6]
		3 administrations every 4 days (days 7, 11, and 15 pi)	C57Bl/6-MOG_35-55_	BD750	MOG_35-55_	LPS at 100 ng/ml for 24 h	Early therapeutic treatment	Delay of disease onset and decreased EAE severity. Reduction of inflammatory infiltrates and demyelination in the CNS. Increase of Treg and reduction of Th1 and Th17 cells in the spleen	One (day 7 pi) or two (day 7 and 11 pi) administrations of antigen-specific tolDCs did not show clinical benefit	[7]
		3 administrations every 4 days (days 19, 23, and 27 pi)	C57Bl/6-MOG_35-55_	BD750	MOG_35-55_	LPS at 100 ng/ml for 24 h	Late therapeutic treatment	No clinical improvement		[7]
		3 administrations every 4 days (days 7, 11, and 15 pi)	C57Bl/6-MOG_35-55_	Tofacitinib	MOG_35-55_	LPS at 100 ng/ml for 24 h	Early therapeutic treatment	Amelioration of clinical signs related with less leukocyte infiltration and demyelination in spinal cord. Reduction in of Th1 and Th17 cells and enhance of Treg in the spleen		[8]
		3 administrations every 4 days (13, 17, and 21 pi)	C57Bl/6-MOG_35-55_	MOG mRNA electroporated-VitD3-tolDCs	MOG_35-55_	LPS at 100 ng/ml for 24 h	Therapeutic treatment	Reduced disease severity related with low number of spinal cord lesions (MRI), decreased MOG_35-55_ T-cell reactivity and less secretion of IL-17, IFN-γ, TNF, and GM-CSF		[9]
		3 administrations every 4 days (14, 18, and 22 pi)	C57Bl/6-MOG_40-55_	VitD3-tolDCs	MOG_40-55_	LPS at 100 ng/ml for 24 h	Therapeutic treatment	Improvement of clinical symptoms. Inhibition of MOG_40-55_ T-cell reactivity, increase of FoxP3+ Treg and IL-10 secretion		[10]
		3 administrations every 4 days (15, 19, and 23p i) for short-term treatment. Extra administrations at days 32, 41, 50, and 68 pi, for long-term treatment	C57Bl/6-MOG_40-55_	VitD3-tolDCs	MOG_40-55_	LPS at 100 ng/ml for 24 h	Therapeutic treatment	Clinical amelioration. Inhibition of MOG_40-55_ T-cell reactivity and induction of FoxP3+ Treg and IL-10 secretion (short-term treatment). Inhibition of MOG_40-55_ T-cell reactivity, increase of Breg and activated NKT cells, and reduction of % and activation of NK cells (long-term treatment)	Cryopreserved VitD3-tolDCs	[11]
		3 administrations every 4 days (13, 17, and 21 pi)	C57Bl/6-MOG_35-55_	VitD3-tolDCs	MOG_35-55_	LPS at 100 ng/ml for 24 h	Therapeutic treatment	Improvement of clinical symptoms by MOG-tolDCs enhanced by MOG-tolDC+IFN-β combined therapy. Inhibition of MOG_35-55_ T-cell reactivity and increase secretion of IL-10	Tolerogenic therapy tested in combination with IFN-β treatment	[3]
NOD model										
Route of administration	Dose	Number of administrations and frequency	Model	tolDC therapy			Type of treatment	Outcome	Observations	Ref
				Tolerogenic agent	Antigen	Maturation				
Intraperitoneal	1 × 10^6^	1 single administration 7 days after disease onset (in combination with 1 insulin unit)	NOD	NIT-1 apoptotic cells	Islet apoptotic bodies	No	Therapeutic	No amelioration of diabetes (determined by assessing insulitis score and by measuring blood glucose levels and C-peptide concentration)	Tolerogenic therapy tested with or without rapamycin treatment (from the day of disease onset). No improvement was observed using rapamycin	[15]
Subcutaneous (abdominal flank, near to pancreas location)	2 × 10^6^	1 single administration or 8 administrations (1 administ/week) w/o insulin co-treatment	NOD	Antisense oligonucleotides targeting mRNA of CD40, CD80 and CD86 costimulatory molecules	No	No	Therapeutic	Stable long-term restoration of pre-diabetic glucose levels, in the absence of any exogenous insulin administration for at least 4 months after cessation of tolDC administration	Multiple injections are required for stable, long-term restoration of blood glucose to levels similar to pre-diabetic conditions	[16]
Subcutaneous (hind footpads)	1 × 10^5^	Short treatment: 3 administrations every week Long treatment: 3 administrations every week + booster administrations every 2 weeks	NOD	Not used (iDC)	Insulin *β*9-23 (DD), GAD65_78-97_ (SD) or GAD65_260-279_ (ID)	No	Early therapeutic	Long-term treatment with iDC pulsed with SD and ID β cell peptides delay T1D onset by expanding and enhancing function of CD4+ FoxP3+ Treg		[17]
Intravenous	1 × 10^6^	1 single administration after the onset of hyperglycaemia	NOD	Electroporation of IL-4 mRNA (eDC/IL-4)	No	No	Therapeutic	Increase of survival in 1/3 of mice treated. Moderate hyperglycaemia and increased of CD25+ FoxP3+ T cells in spleen		[18]
							Early therapeutic	Reduction of diabetes incidence		[18]
CIA model										
Route of administration	Dose	Number of administrations and frequency	Model	tolDC therapy			Type of treatment	Outcome	Observations	Ref
				Tolerogenic agent	Antigen	Maturation				
Intravenous	2 × 10^5^	1 single administration in mice with established CIA (score = 2)	CIA	VIP	CII	LPS at µg/ml for 48 h	Therapeutic	Abrogation of clinical disease progression. Inhibition of auto-antigen proliferation IFN-γ secretion, reduction of CII autoantibodies and expansion of IL-10/TGFβ-producing Treg	Similar results in the EAE model	[23]
	1 × 10^6^	1 single administration at day 32 (4 days after LPS booster)	CIA	DC/IL-4 (genetically modified to express IL-4)	No	No	Therapeutic	Decrease in disease severity. Reduction in number and swelling of arthritic paws and joints with less inflammation and bone erosion		[24]
			CIA	DC/FasL (iDC genetically modified to overexpress FasL)	No	No	Therapeutic	Reduced disease severity. Decrease in number of inflammatory cells, reduction of synovitis and less cartilage destruction in joints. Inhibition of T-cell proliferation and IFN-γ secretion after CII stimulation	A single dose of DC/FasL confers a long-term therapeutic effect	[26]
		1 single administration at day 28 (7 days after CII booster)	CIA	siRNA-BAFF DC	CII	LPS at 1 µg/ml for 24 h	Therapeutic	Clinical amelioration (reduction of arthritis index and swollen joint count). Decrease of synovial hyperplasia, cartilage erosion, and inflammation mediated by induction of CD25+ FoxP3+ Treg and inhibition of Th17 cells		[27]
	1 × 10^4^, 1 × 10^5^, 1 × 10^6^	3 administrations at days 27 (6 days after booster), 31, and 35	CIA	Tacrolimus	CII	LPS (1 µg/ml) + IFN-γ (1000 U/ml) for 24 h	Therapeutic	Doses of 1 × 10^4^ and 1 × 10^5^ cells did not improve CIA severity. Dose of 1 × 10^6^ delayed disease onset and reduced disease severity	One single administration of 1 × 10^6^ cells was more effective than three doses of tolDCs suppressing CIA in the late phase of the disease	[28]
	1 × 10^6^	1 single administration at day 27 (6 days after booster)					Therapeutic	Strong and long-term reduction of disease severity		
	1 × 10^4^, 2 × 10^5^, 1 × 10^6^ and 2.5 × 10^6^	3 administrations at days 3, 7, and 11 (3 days after disease onset)	CIA	Dexa+VitD3	CII	LPS at 0.1 µg/ml LPS for 16 h	Therapeutic	Reduction of disease severity and progression of arthritis in relation with decrease of Th17 cells and increase of IL-10 producing T cells. At least 3 i.v. injections of 1 × 10^6^ tolDCs are needed for an optimal therapeutic effect	I.v. injection of more than 1 × 10^6^ cells did not improve the therapeutic effect. 1 single i.v. administration of 1 × 10^6^ cells or 3 i.p. injections did not induce beneficial effect	[29]
Intraperitoneal	5 × 10^5^	1 single administration at day 35 (14 days after CII booster)	CIA	Short-term LPS stimulation	CII	LPS at 1 µg/ml LPS for 4 h	Therapeutic	Amelioration of CIA clinical symptoms. Reduction of inflammatory infiltrates and joint damage	2 doses of 4 hLPS/CII/DC at days 28 and 35 did not induce higher improvement	[30]
	2 × 10^6^	1 single administration at day 40 (after disease onset)	CIA	Bay 11-7082 or VIP	No	LPS (10 ng/ml) + TNF (10 ng/ml)	Therapeutic	Reduced disease severity. Decreased synovial hyperplasia, bone erosion, and inflammation	Similar clinical effect of VIP‐DC and Bay 11‐7082‐DC treatment, but VIP-DC may exert a better preventive effect on bone destruction	[31]
Subcutaneous	5 × 10^5^	1 single administration at day 6 post-induction	Monoarticular AIA	Bay 11-7082	mBSA	No	Therapeutic	Clinical disease suppression in a IL-10 dependent way		[32]
	2 × 10^5^	2 administrations at day 21 (after CII booster) and day 29	CIA	T74	CII	LPS at 1 µg/ml for 4 h	Early therapeutic	Clinical amelioration. Reduced joint inflammation, joint deformation, and bone erosion. Increase of Treg		[33]
	2 × 10^5^	2 administrations at day 21 (after CII booster) and day 29	CIA	Rosiglitazone	CII	LPS at 1 µg/ml for 24 h	Early therapeutic	Abrogation of disease progression. Reduction of inflammation and increase of Treg		[25]

AIA: antigen-induced arthritis; CIA: Collagen-induced arthritis; DD: Dominant determinants; ID: Ignored determinant; mBSA: methylated bovine serum albumin; SD: Subdominant determinant; T74: ((E)-1-(3-Aminophenyl)-3-(2,5-dimethoxyphenyl)prop-2-en-1-one), TGF-β signalling agonist; TNF: tumour necrosis factor‐α; VIP: vasoactive intestinal peptide.

**Table 2.** Clinical trials using tolDC treatment in autoimmune diseases

Multiple Sclerosis										
Route of administration	Dose	Number of administrations and interval of dose	tolDC therapy			Number of patients	Phase	Study status	Outcome	NCT number
			Tolerogenic agent	Antigen	Maturation					
Intravenous	Dose escalation study using 50, 150, and 300 ×. 10^6^ tolDCs/injection	3 doses every 2 weeks	Dexa	Pool of 7 myelin peptides for MS + AQP4_63-76_ for NMOSD	Maturation cocktail of IL-1β, IL-6, TNF-α, and PGE2 for 24 h	8 RRMS and progressive MS patients and 4 NMOSD patients	Ib	Completed	Treatment was safe and well tolerated. Increase production of IL-10 following peptides stimulation and reduced memory CD8+ T cells and memory NK cells at week 12 (compared with baseline) [12]	NCT02283671 TolDec-EM-NMO
	NR	Combination therapy of low-to-moderate efficacy immunomodulatory drugs (IFN-β, glatiramer acetate, teriflunomide or dimethyl fumarate + 3 doses every 2 weeks of tolDCs)	Dexa	Pool of 7 myelin peptides	Maturation cocktail of IL-1β, IL-6, TNF-α, and PGE2 for 24 h	45 RRMS patients	II	Recruiting	–	NCT04530318 TolDecCOMBINEM
Intradermal	Dose escalation study using 5, 10, and 15 × 10^6^ tolDCs/injection	A total of 6 administrations: 4 doses every 2 weeks + 2 doses every 4 weeks	VitD3	Pool of 7 myelin peptides	Maturation cocktail of TNF+IL1-β+PGE2 for 24 h	9 active MS patients	I	Active, not recruiting	NR	NCT02618902 MS-tolDCs [13]
Intranodal	Dose escalation study using 5, 10, and 15 × 10^6^ tolDCs/injection + an additional cohort of patients treated with the highest dose of well tolerated tolDCs + IFN-β treatment	A total of 6 administrations: 4 doses every 2 weeks + 2 doses every 4 weeks	VitD3	Pool of 7 myelin peptides	Maturation cocktail of TNF+IL1-β+PGE2 24 h	12 active MS patients	I	Recruiting	–	NCT02903537 TOLERVIT-MS [13]
Type 1 diabetes										
Route of administration	Dose	Number of administrations and interval of dose	tolDC therapy			Number of patients	Phase	Study status	Outcome	NCT number
			Tolerogenic agent	Antigen	Maturation					
Intradermal	10 million tolDCs/administ	4 administrations every 2 weeks	Antisense DNA oligonucleotides targeting mRNA of CD40, CD80, and CD86	No	No	10 (7 tolDCs + 3 control DCs)	I	Completed	Treatment was safe and well tolerated. Increase of B220+ CD11c-B cells [19]	NCT00445913
						24 newly diagnosed T1D patients	II	Unknown	NR	NCT02354911
	Dose escalation study using 5, 10, and 20 × 10^6^ tolDCs/administ	2 administrations: day 0 and day 28	VitD3+Dexa	Proinsulin Peptide (C19-A3)	GM-CSF, 800 U/ml; IL-1β, 1600 U/ml; IL-6, 500 U/ml; TNFα, 335 U/ml and PGE2 (2 µg/ml) for 48 h	9 patients with long-lasting T1D	I	Completed	Treatment was feasible and safe [20]	NCT04590872 PIpepTolDC D-Sense trial
Rheumatoid arthritis										
Route of administration	Dose	Number of administrations and interval of dose	tolDC therapy			Number of patients	Phase	Study status	Outcome	NCT number
			Tolerogenic agent	Antigen	Maturation					
Intradermal	Low-dose (0.5 to 1 × 10^6^ cells) and high dose (2 to 4.5 × 10^6^ tolDCs)	1 single injection	Bay 11-7082 (NF-KB inhibitor)	Citrullinated peptides from fibrinogen α and β chains, vimentin and collagen type II	No	18 RA patients carrying HLA-DRB1 SE alleles	I	Completed	Treatment was safe. Decrease of effector T cells and reduction of proinflammatory cytokines and chemokines at 1 month after treatment [63]	Rheumavax
Subcutaneous	Low-dose (5 × 10^6^ tolDCs) and high dose (15 × 10^6^ tolDCs)	5 administrations (every 2 or 4 weeks)	NR	PAD4, RA33, citrullinated-filaggrin and vimentin	NR	12	I	Completed	Treatment was safe and well tolerated. Reduction of antigen-specific autoantibodies [37]	CRiS KCT0000035 CreaVax-RA
Intraarticular injection (into the knee join)	Dose escalation study using 1, 3, 5, 8, and 10 × 10^6^ tolDCs	1 single administration	Dexa (differentiation in presence of IFN-α/GM-CSF)	No	Azoximer bromides	12	I	Completed	Treatment was safe, well tolerated, and had a potential for long-term efficiency [38]	NCT03337165 TolDCfoRA
	Dose escalation study using 1, 3, and 10 × 10^6^ tolDCs	1 single administration	Dexa + VitD3	Autologous synovial fluid	MPLA (1μg/ml) for 20 h	9 RA patients with inflammatory arthritis	I	Completed	Treatment was safe, feasible and acceptable for patients [39]	NCT01352858 AuToDeCRA
Intranodal (inguinal lymph node)	Dose escalation study using low, intermediate and high dose: 5, 10, and 15 × 10^6^ tolDCs/injection	2 administrations every 4 weeks	Dexa + VitD3	B29-peptide of HSP70	MPLA (1 μg/ml) for 20 h	18	I/II	Recruiting	–	NCT05251870, TOLERANT

Dexa: Dexamethasone; MPLA: monophosphoryl lipid A; NMOSD: neuromyelitis optica spectrum disorders; NR: not reported; PGE2: prostaglandin E2; RRMS: relapsing-remitting multiple sclerosis patients.

### Multiple sclerosis

MS is a neurodegenerative disease of the central nervous system (CNS) caused by autoimmune attack and destruction of myelin sheaths producing different motor, visual and sensory alterations. EAE is the most used animal model of MS since it reproduces most of the clinical and histopathological features of patients with MS. EAE is induced in C57Bl/6 mice by administering peptide 35–55 of the myelin oligodendrocyte glycoprotein (MOG_35-55_) emulsified with complete Freund adjuvant plus an additional injection of pertussis toxin (Day 0 and 2 post-induction). At Days 10-12 post-immunization, mice initiate a chronic non-remitting clinical course of the disease consisting of ascendent paralysis.

Many studies using tolDC therapy in EAE have been tested in recent years. Table 1 shows data from studies performed from 2015 to 2022 using early therapeutic (initiation of treatment close to the disease onset day) or therapeutic approaches (first dose of tolDCs administered after the onset of clinical symptoms). The preventive approach (administration of tolDCs before immunization) was excluded from the analysis. As shown in Table 1, all preclinical EAE studies were performed using antigen-specific tolDCs (MOG-tolDCs) injected by i.v. administration [1–11], probably because of the difficulty of injecting tolDCs directly into the affected tissue, the CNS (brain and spinal cord), and with the objective of restoring immune tolerance through peripheral lymphoid organs. Multiple tolDC administrations are required to induce a long-lasting clinical effect in EAE [11], and with the exception of the study of Li *et al*. using a single administration in an early therapeutic treatment, all the preclinical studies reported in this model applied three administrations of 0.3, 0.8, or 1 million MOG-tolDCs every 3 or 4 days (Table 1). The results from studies performed in mice showing clinical symptoms of the disease (therapeutic approach) showed promising abrogation of disease progression following MOG-tolDC treatment that was mediated mainly by Treg and Breg induction, increased IL-10 production and reduced MOG-specific T-cell proliferation [3, 9–11]. Notably, late therapeutic treatment did not achieve clinical amelioration [7].

Currently, three phase I clinical trials have been conducted in MS patients (Table 2). In the first trial carried out in eight either relapsing-remitting or progressive MS (and four neuromyelitis optica spectrum disorders) patients, Dexa-tolDCs loaded with a pool of seven myelin peptides and one peptide of aquaporin-4 (AQ4_63-76_) were injected i.v. three times every 2 weeks in a dose-escalation design (50, 150, and 300 million tolDCs). Clinically, peptide-loaded Dexa-tolDC treatment was safe and well tolerated, and immunoregulatory mechanisms induced by the tolerogenic therapy were suggested (such as increased production of IL-10 and reduction in memory CD8+ T cells and NK cells) (Table 2 [12]). However, a technical limitation to accomplishing the highest dose (300 million) of tolDCs was reported. Currently, a phase II clinical trial administering three doses of myelin-specific Dexa-tolDCs i.v. every 2 weeks is recruiting MS patients for receiving a combined treatment of tolDCs plus a low-to-moderate efficacy immunomodulatory drug (TolDecCOMBINEM) (Table 2). In contrast to the i.v. administration, another two coordinated phase I clinical trials for active MS patients have been conducted by injecting 5, 10, and 15 million VitD3-tolDCs loaded with a pool of seven myelin peptides using intradermal (i.d.) (MS-tolDCs) or intranodal (i.n.) (TOLERVIT-MS) delivery. Patients received a total of six administrations of peptide-loaded VitD3-tolDCs, the first four every 2 weeks and the last two every 4 weeks (Table 2 [13]). Preliminary results indicated that i.d. and i.n. delivery of myelin-specific VitD3-tolDCs are safe, feasible, and well tolerated by active MS patients [14].

### Type 1 diabetes

T1D is an autoimmune disease caused by the destruction of pancreatic insulin-producing β cells. Because of their insulin deficiency, patients suffer from a complex metabolic derangement that needs to be managed with exogenous ­insulin administration. The NOD mouse model is a spontaneous model sharing genetic, immunological, and environmental similarities with T1D patients. NOD mice develop autoantibodies and autoreactive T cells, causing β cell destruction and hyperglycaemia (disease onset) at approximately 12 and 15 weeks of age. Because of the difficulty in anticipating the onset day of the disease for each animal, most of the studies using tolDCs have been performed using a prophylactic approach. Only four studies administering tolDCs in an early therapeutic or curative approach have been reported (Table 1 [15–18]). The study from Pujol-Autonell *et al*. using intraperitoneal (i.p.) administration of tolDCs loaded with islet apoptotic bodies did not achieve clinical amelioration, probably because the treatment was initiated late (7 days after disease onset) and only a single administration was performed [15]. In contrast, i.v. and subcutaneous (s.c.) (near the pancreas) administration of 1 and 2 million unpulsed genetically manipulated tolDCs (DCs overexpressing IL-4 or downregulating the expression of costimulatory molecules, respectively) restored pre-diabetic glucose levels; importantly, multiple injections were required for stable long-term clinical effects [16, 18].

The first clinical trial with therapeutic tolDCs in T1D patients was carried out by Giannoukakis *et al*. They injected (i.d.) 10 million unpulsed genetically engineered tolDCs (DCs treated with antisense oligonucleotides downmodulating the expression of CD40, CD80, and CD86 costimulatory molecules) in T1D patients with insulin-requiring diabetes for at least 5 years between the time of clinical diagnosis and the first tolDCs injection. Patients received a total of four i.d. administrations every 2 weeks in the abdominal wall above the physical location of the stomach and pancreas. The results showed that treatment was safe and well tolerated, and an increase in B220+ CD11c- B cells was found [19]. Currently, a phase II trial is ongoing to evaluate the efficacy of these unpulsed genetically engineered tolDCs in newly diagnosed T1D patients (NCT023544911, Table 2). In addition, a phase I trial in T1D patients receiving VitD3+Dexa-tolDCs loaded with the proinsulin peptide C19-A3 has been conducted (PIpepTolDCs, D-Sense trial). The treatment of long-duration T1D patients with two i.d. administrations of 5, 10, and 20 million C19-A3-loaded tolDCs was safe and feasible, and no signs of systemic immune suppression were detected [20]. Interestingly, immunological studies revealed that C19-A3-tolDC vaccine was able to control autoimmunity, after 6 months, in three patients exhibiting pre-existing vaccine peptide response by reducing antigen-specific proliferation and interferon-gamma (IFN-γ) production or increasing IL-10 secretion. Regarding glycemic control, all patients showed long standing but well-controlled T1D, together with a long-lasting (up to 3 years) decline in autoimmune response after receiving C19-A3-tolDC treatment [21]. A future phase II trial in recently diagnosed T1D patients with preserved C-peptide production needs to be planned to assess the efficacy of C19-A3-loaded tolDC therapy to delay or halt progressive loss of β-cell function. Finally, a phase I/II clinical trial administering VitD3-tolDCs differentiated in the presence of mesenchymal stem cells is planned to be initiated (NCT05207995). No information about the administration route to be used has been reported.

### Rheumatoid arthritis

RA is a chronic and systemic inflammatory disease characterized by pain, swelling, and stiffness in the joints, although patients can also experience other systemic signs and symptoms [22]. CIA is the most common animal model that shares immunological and pathological features with RA patients. It is induced in mice by immunization with type II collagen (CII) emulsified with complete Freund adjuvant. Approximately 3 weeks post-immunization, an additional administration of CII is performed as a booster of the disease, and alternatively, synchronization can be induced by LPS injection. Different delivery routes have been used to administer tolDCs therapeutically in CIA mice: i.v., i.p., and s.c. (Table 1 [23–33]), with all of them showing a beneficial clinical effect. Most of the therapeutic studies carried out in CIA mice using i.v. delivery of tolDCs administered a single dose of cells (Table 1). In fact, Ren *et al*. reported that a single administration of 1 million CII-tacrolimus-tolDCs through an i.v. route was more effective than three doses of these tolDCs in suppressing CIA [28]. In contrast, Stoop *et al*. reported that three i.v. injections of Dexa-VitD3-tolDC were required for an optimal therapeutic effect [29]. Interestingly, both studies reported that i.v. injection of less than 1 million tolDCs did not provide clinical amelioration. Therefore, preclinical data in the CIA model indicate that i.v. administration of 1 to 3 million tolDCs, depending on the tolDC type, could be sufficient to abrogate disease progression. Moreover, the study of Stoop *et al*. also demonstrated that changing the route of Dexa-VitD3-tolDC administration from i.v. to i.p. (three doses of 1 million and 2.5 million, respectively) abolished the beneficial effect of the therapy [29]. In contrast, Salazar *et al*. demonstrated that a single i.p. dose of 5 × 10^5^ tolDCs generated by short-term stimulation with LPS was able to reduce clinical severity, and this effect was not increased by administering two i.p. doses of cells [30]. Similarly, therapeutic treatment with Bay 11-7082- and vasoactive intestinal peptide-induced tolDCs ameliorated clinical signs after a single i.p. administration of 2 million tolDCs [31]. Regarding s.c. delivery of tolDCs, there are no studies comparing multiple versus a single administration of tolDCs. In this context, clinical amelioration occurred after a single s.c. dose of 5 × 10^5^ tolDCs (Bay 11-7082-induced tolDCs [32]) and following two s.c. doses of 2 million tolDCs (T74- and rosiglitazone-induced tolDCs [25, 33]). Finally, although CIA is the most common animal model used to study RA, beneficial effects of antigen-specific tolDC therapy in the proteoglycan-induced arthritis (PGIA) animal model have been also reported [34, 35].

A total of four phase I clinical trials analysing the safety and tolerability of tolDC treatment in RA patients have been conducted (Table 2 [36–39]) using different delivery routes. In the first trial (Rheumavax), enrolled RA patients received a single administration of 1 million tolDCs (citrullinated peptide pool-loaded Bay 11-7082-induced tolDCs) through i.d. delivery (Table 2 [36]). The results showed that the treatment was safe, and some immunomodulatory mechanisms of action were found (reduction in effector T cells and proinflammatory cytokines) [36]. Because RA patients suffer from joint inflammation, intraarticular (i.a.) administration (into the knee joint) was chosen in two trials [20, 38]. Patients from both trials, AuToDeCRA and TolDCfoRA, received a single administration of autologous synovial fluid-loaded Dexa+VitD3-tolDCs or unpulsed Dexa-tolDCs, respectively, in a dose-escalation design (from 1 to 10 million tolDCs). Both treatments were safe, feasible, and well tolerated. Currently, the second AuToDeCRA clinical trial is in preparation, in which Dexa-VitD3-tolDCs will be loaded with a cocktail of citrullinated autoantigens and injected via three different routes: i.n., i.d., and i.a. Regarding s.c. tolDC delivery, the CreaVax-RA trial reported that five administrations (every 2 or 4 weeks) of 5 and 15 million tolDCs loaded with a pool of peptides were safe and well tolerated and reduced antigen-specific autoantibodies (Table 2 [37]). Finally, a dose-escalation phase I/II trial in RA patients receiving two i.n. injections (into the inguinal lymph nodes) of 5, 10, and 15 million Dexa+VitD3-tolDCs loaded with B29-HSP70 peptide is now recruiting patients (TOLERANT).

## Pros and cons of tolDC administration routes

Studies in non-human primates reported that i.v. administration is the most tolerogenic route of administration [40]. Moreover, in terms of feasibility, i.v. administration is the most convenient delivery route of human tolDCs and is the most common route of administration when the target tissue is not easily accessible. However, because i.v. delivery triggers tolDCs biodistribution throughout the body, the number of cells required to facilitate migration to secondary lymphoid organs is likely to be higher than with other routes of administration. Following these recommendations, i.v. injection of tolDCs could be the most convenient delivery route for MS patients since the target tissue experiencing autoimmune attack is the CNS. However, the dose-escalation TolDec-EM-NMO trial reported a technical limitation for i.v. ­administration of the highest tolDC dose (300 million myelin-Dexa-tolDCs), making this approach less feasible. To solve this problem, most of the phase I clinical trials reported to date have injected tolDCs directly into the affected tissue (if it is accessible) or i.d., in proximity to the target organ. For example, two trials in RA patients, TolDCfoRA and AutoDeCRA, performed i.a. (into the knee joint) injection of tolDCs; in the MS-tolDC study, cells were administered to MS patients i.d. near the cervical lymph nodes; and finally, two clinical trials conducted in T1D patients used i.d. injections in the abdominal region near the pancreas (NCT00445913 and PIpepTolDC). The main concern about i.d., s.c., and i.p. administration is the capability of tolDCs to migrate and reach draining lymph nodes or inflamed tissues, since tolDCs express low levels of CCR7. Consequently, direct i.n. injection of tolDCs has been proposed as a solution for the low migratory potential of tolDCs. In fact, myelin-VitD3-tolDCs have been injected into the cervical lymph nodes of MS patients as a well-tolerated delivery method for MS patients. However, highly trained personnel were necessary for performing echography-guided tolDC i.n. injection (TOLERVIT-MS).

## Lessons learned from delivery of immunogenic DCs for cancer treatment

In the context of cancer immunotherapy, autologous DCs maturated and loaded *ex vivo* with tumour antigens are a therapeutic strategy to induce long-lasting tumour-specific T-cell responses and are being evaluated in a wide variety of trials for cancer patients [41, 42].

Although the optimal route for DC vaccination is also a matter of debate in cancer therapy, the study of Verdijk *et al*. revealed that following i.d. vaccination of immunogenic DCs in melanoma patients, only up to 4% of DCs migrate to adjacent lymph nodes, whereas after i.n. injection, migration of up to 84% DC was observed [43]. Nevertheless, it has been found that after i.n. administration, DC migration to adjacent lymph nodes was achieved only when DCs were correctly injected into the lymph node. The study of de Vries in 2005 reported that correct i.n. administration was accomplished only in approximately 50% of vaccinations, although cells were injected by a highly experienced radiologist under ultrasound guidance [44]. Interestingly, Lesterhuis *et al*. reported that despite the elevated DC redistribution to adjacent lymph nodes following i.n. injection in melanoma patients; i.d. administration of immunogenic DCs resulted in superior antitumour T-cell induction [45]. Collectively, these data suggest that i.d. administration of tumour-specific DCs is easier and more effective than i.n. injection for the treatment of melanoma and cancer patients. However, in the context of tolerance induction, i.n. administration could be more convenient than i.d. tolDC injection. In agreement with this argument, it has been found that within 48 h post-i.n. injection, large numbers of apoptotic DCs are cleared by anti-inflammatory CD163 macrophages infiltrating lymph nodes, which may trigger an immunoregulatory response [43, 45].

Finally, a meta-analysis of 231 prostate cancer and 172 renal cell cancer patients revealed that vaccination routes with access to draining lymph nodes (i.d., i.n., intralymphatic, and s.c.) resulted in better clinical response rates in comparison to direct i.v. injection [42], probably because mature DCs do not express the homing receptor CD62L necessary to enter lymph nodes across high endothelial venules from blood [42]. Indeed, murine studies in cancer and EAE have shown that i.v. administration of immunogenic DCs and tolDCs accumulate mainly in the spleen, lungs and liver but not in lymph nodes [10, 46].

## *In vivo* tracking of therapeutic tolDCs

It is known that the route of administration influences tolDCs biodistribution, therefore affecting their migration to the desired tissue and influencing the number of cells required to be injected. In this regard, we have reported that after i.v. administration of 1 million antigen-specific bone marrow-derived VitD3-induced tolDCs labelled with CellVue NIR815 dye (MOG-tolDC-NIR815) to EAE animals, cells immediately accumulated in the lungs [10]. After 24 h, MOG-tolDC-NIR815 was highly concentrated in the liver and moderately high in the spleen, where the cell-labelled signal increased after 48 h and remained elevated until Day 7 post-injection (and was detected even 14 days after administration). Moreover, after systemic administration of MOG-tolDC-NIR815, cells were also detected at low concentrations in other tissues, such as the thymus, lymph nodes, brain, kidneys, and bone marrow, meaning that following i.v. injection, cells were biodistributed throughout the body, but they were concentrated in the spleen, where they most likely exerted their tolerogenic effect. Clinical results revealed a reduction in disease severity, thus indicating that antigen-specific tolDCs do not need to cross the blood‒brain barrier to reach the affected tissue to perform their tolerogenic function. Similarly, Kim *et al*. showed that following i.v. administration of PKH26 fluorescent-labelled or luciferase-transduced iDCs genetically modified to express IL-4 in animals with CIA, cells were found only in the liver, spleen, and lymph nodes at 6 h or 24 h post-injection [24]. Similarly, Stoop *et al*. reported that i.v. injected Dexa-VitD3 tolDCs migrated to the lung, spleen, liver, feet, and draining lymph nodes [29]. Together, these results suggest that after systemic administration of tolDCs, cells migrate to the lymphoid organs, where they modulate T-cell responses.

Although it has not been conclusively demonstrated, a generally ­accepted dogma is that tolerance induction occurs in the lymphoid organs. Indeed, murine studies have shown that tolerance to harmless inhaled or ingested antigens requires CCR7-mediated migration of antigen-carrying DCs to the draining lymph nodes [47, 48]. The same has been reported in murine DC vaccination studies for cancer; the induction of optimal antitumour T-cell responses correlates with the number of antigen-loaded DCs reaching the draining lymph node [49, 50]. Therefore, it is reasonable to hypothesize that therapeutic tolDCs need to exert their regulatory actions in ­lymphoid tissue, where they can interact with naive T cells and induce antigen-specific regulatory T cells. However, it cannot be excluded that tolDCs could also have beneficial effects in the diseased target tissue, either through the induction of anergy in memory T cells and/or through the secretion of anti-inflammatory molecules that can dampen inflammation locally, such as IL-10 or TGF-beta [51, 52].

Most tolDC clinical trials to date have been carried out with monocyte-derived DCs (moDCs). From studies in the cancer field, it is known that only low numbers of mature moDCs reach the draining lymph node after i.d. administration in humans (under 5%) [43], although this low migration rate is sufficient to induce antitumour T-cell responses. Nevertheless, the inherent low migratory ability of moDCs may pose a problem for therapeutic tolDCs, as these cells are likely to have even lower migratory ability than mature moDCs due to the immunomodulatory agents used to generate these cells. For example, compared to mature moDCs, tolDCs generated with Dexa and the active form of VitD3 have substantially reduced expression of CCR7 and therefore a limited capacity to migrate in a CCR7-dependent manner [53]. Indeed, these tolDCs failed to migrate towards the draining lymph node after administration into an inflamed knee joint of a RA patient, as determined by imaging of ^111^Indium-labelled tolDCs [54] This observation possibly explains the local but not systemic effects of tolDC administration [39]. It is therefore crucial to improve our understanding of the biodistribution of tolDCs after administration via different routes and how this relates to their immunomodulatory actions. *In vivo* tracking of tolDCs by non-invasive imaging would be the ideal method to provide novel insights into the location and migration of these cells after injection. *In vivo* tracking not only would help in determining the optimal injection route but also would be a useful tool for further optimization of tolDC products in terms of their tissue-homing ability. In addition, *in vivo* tracking could serve as a biomarker to confirm successful administration/migration of tolDCs into the desired tissue.

Currently, the most comprehensive method for DC imaging in humans is magnetic resonance imaging (MRI) (recently reviewed in [55]), not only because of its excellent soft tissue contrast and high spatial resolution but also because of pragmatic reasons, such as the availability of clinically approved MRI labelling agents that are suitable for DCs and the wide availability of clinical MRI scanners. Both ^1^H and ^19^F clinical MRI have been employed for *in vivo* tracking of DCs in cancer vaccines [44, 56], with ^19^F-MRI having the advantage that it detects the ^19^F nucleus, which is highly MR sensitive compared to most other MR-visible nuclei, and there is a negligible endogenous ^19^F signal *in vivo*. Both approaches require *ex vivo* labelling of DCs, with superparamagnetic iron oxides (SPIOs) being the most used for ^1^H MRI and perfluorocarbon for ^19^F-MRI.

In the tolDC field, we (Hilkens and colleagues) have recently tested the labelling of therapeutic human tolDCs with nanoparticles containing ^19^F (^19^F-NP) for detection by ^19^F-MRI [54]. We found that tolDCs readily endocytosed ^19^F-NP with acceptable effects on cell viability and yield and, importantly, without affecting the tolerogenic features of the cells. The MRI signal-to-noise ratios obtained were estimated to allow the detection of approximately 150 000 ^19^F-labelled tolDCs (using a 3 Tesla scanner), although a number of caveats likely enhance the detection limit *in vivo,* e.g. tolDC location and dispersal. While it is likely that these tolDC numbers can be detected in the skin or in tissues close to the skin (e.g. superficial lymph nodes), the detection of these cells in deeper tissues will pose more of a challenge. In addition, the MRI signal will be negatively impacted by the dispersal of the cells, e.g. through the cells spreading out locally, at the primary injection site, or through cell migration to secondary sites. This also means that the likelihood of tolDC detection at secondary sites is low, as it would require a substantial cell density to enable detection by ^19^F-MRI. Indeed, Ahrens *et al*. have shown that although ^19^F-labelled human mature moDCs could be detected at the primary injection site (the dermis) and the signal diminished over time, no migration of any DC to, e.g. the draining lymph node could be observed [56]. Despite this limitation, there remains value in imaging tolDCs after administration. First, verifying that the cells have been injected correctly carries considerable importance, as incorrect injection may hinder the ability of DCs to reach the desired target tissue. For example, this is the case for intranodal injections, which can fail if the injection is not accurate and/or if there is a backflow of the cells. Indeed, an elegant study by de Vries *et al*. [44] showed that accurate delivery of mature moDCs into the lymph node under ultrasound guidance by an experienced radiologist was achieved in only ~50% of cases and that subsequent migration of these cells could be observed only if the cells had been injected correctly. Therefore, the accuracy of injection could be one of the factors that explains the variable immunological and clinical responses in DC-treated patients. Second, it will be of interest to monitor the ‘efflux’ of tolDCs after injection, which could indicate cell migration and/or the clearance of dead tolDCs by tissue macrophages.

A major limitation of any of the current clinical imaging methods is that the cells need to be labelled with an imaging agent. First, it cannot be excluded entirely that the labelling process does not affect some (known or unknown) functional features of the cells. It has been reported, e.g. that SPIO labelling of murine DCs can reduce their migratory ability *in vivo* [57]. Second, the imaging agent could be taken up by other phagocytic cells within the tissue, either after leaking from the cells or by clearing labelled dead cells. This could potentially lead to the misinterpretation of imaging data. Label-free or indirect-labelling imaging techniques would therefore be the preferred option. One possible approach is the use of chemical exchange saturation transfer MRI, which was recently applied for the label-free tracking of mesenchymal stem cells in a mouse model [58]. Another option would be to use reporter gene-based molecular imaging [59]. In this approach, cells are genetically engineered to ectopically express a reporter gene; the cells can then be detected *in vivo* following the administration of a suitable molecular probe (a radioisotope with a short half-life) that targets the reporter. This can be performed repeatedly, enabling the tracking of viable cells over time. This approach has already been successfully applied to Tregs and CAR-T cells in mouse models [60, 61], and it would be of considerable interest to develop this imaging technology for tolDCs. The only caveat is that tolDCs would have to be genetically engineered, but this raises the opportunity to include other genes of interest; an engineering strategy that combines reporter gene expression (for molecular imaging) and, e.g. CCR expression (e.g. CCR7 for enhanced lymph node homing potential) would for certain constitute a major advancement in the tolDC field. For example, it has been demonstrated that introducing mRNA encoding CCR5 by electroporation in VitD3-tolDCs enhanced their capacity to transmigrate in a chemokine gradient *in vitro* [62].

## Questions to be addressed in future studies

Currently, data collected from studies using tolDC therapy in autoimmune disorders do not provide enough evidence to define the most appropriate route of tolDC administration. In this context, important concerns about other parameters, such as the dose, timing, and frequency of tolDC administration, need to be solved to establish the best route for tolDC administration. For example, since the optimal cell number for tolDC treatment has not been determined for any of the available delivery routes, it is possible that the doses used in clinical trials were not adequate to induce potent clinical effects. In this regard, is important to mention the relevance of monitoring changes on antigen-specific reactivity, cytokine profile, lymphocyte subpopulations, autoantibodies levels, etc. to determine efficacy of the treatment in early phase of clinical trials.

Novel strategies for *in vivo* tolDC imaging will help in the understanding of the biodistribution and accumulation of tolDCs and to define the most effective way to use tolDC therapy in the treatment of autoimmune diseases.

## Abbreviations

AQ4: aquaporin-4
CII: II collagen
CIA: collagen-induced arthritis
CNS: central nervous system
DCs: dendritic cells
Dexa: dexamethasone
EAE: experimental autoimmune encephalomyelitis
i.a.: intraarticular
i.d.: intradermal
iDCs: immature DCs
i.n.: intranodal
i.p.: intraperitoneal
i.v.: intravenous
LPS: lipopolysaccharide
MOG: myelin oligodendrocyte glycoprotein
MPLA: monophosphoryl lipid A
MRI: magnetic resonance imaging
MS: multiple sclerosis
NOD: nonobese diabetes
PGIA: proteoglycan-induced arthritis
PGE2: prostaglandin E2
RA: rheumatoid arthritis
s.c.: subcutaneous
T1D: Type 1 diabetes
tolDCs: tolerogenic DCs
VIP: vasoactive intestinal peptide
VitD3: vitamin D3
